# Seasonal Dynamics of Soil Fungal and Bacterial Communities in Cool-Temperate Montane Forests

**DOI:** 10.3389/fmicb.2019.01944

**Published:** 2019-08-23

**Authors:** Nobuhiko Shigyo, Kiyoshi Umeki, Toshihide Hirao

**Affiliations:** ^1^The University of Tokyo Chichibu Forest, Graduate School of Agricultural and Life Sciences, The University of Tokyo, Chichibu, Japan; ^2^Graduate School of Horticulture, Chiba University, Matsudo, Japan

**Keywords:** forest ecosystems, model-based clustering, soil bacteria, soil fungi, temporal dynamics

## Abstract

Both fungal and bacterial communities in soils play key roles in driving forest ecosystem processes across multiple time scales, but how seasonal changes in environmental factors shape these microbial communities is not well understood. Here, we aimed to evaluate the importance of seasons, elevation, and soil depth in determining soil fungal and bacterial communities, given the influence of climate conditions, soil properties and plant traits. In this study, seasonal patterns of diversity and abundance did not synchronize between fungi and bacteria, where soil fertility explained the diversity and abundance of soil fungi but soil water content explained those of soil bacteria. Model-based clustering showed that seasonal changes in both abundant and rare taxonomic groups were different between soil fungi and bacteria. The cluster represented by ectomycorrhizal genus *Lactarius* was a dominant group across soil fungal communities and fluctuated seasonally. For soil bacteria, the clusters composed of dominant genera were seasonally stable but varied greatly depending on elevation and soil depth. Seasonally changing clusters of soil bacteria (e.g., *Nitrospira* and *Pelosinus*) were not dominant groups and were related to plant phenology. These findings suggest that the contribution of seasonal changes in climate conditions, soil fertility, and plant phenology to microbial communities might be equal to or greater than the effects of spatial heterogeneity of those factors. Our study identifies aboveground–belowground components as key factors explaining how microbial communities change during a year in forest soils at mid-to-high latitudes.

## Introduction

Learning about the temporal patterns and processes of microbial communities can help us understand the drivers of community stability and ecosystem functioning (Shade et al., 2012). Some studies have found that temporal dynamics can be identified in microbial communities as well as other biological communities (Faust et al., 2015; Buscardo et al., 2018). Indeed, the temporal dynamics of microbial communities has been observed across different time scales: rapid responses associated with dissolved organic matter within minutes (Fenchel, 2002), seasonal periodicity (Gilbert et al., 2012), and succession over several years or decades relating to growth and development of host organisms (Koenig et al., 2011; Clemmensen et al., 2015). Among them, clarifying the seasonal dynamics of soil microbial communities is of particular importance for improving ecosystem management policy, as well as understanding microbial community assembly. In mid-to-high latitude areas, seasonal climatic events drive forest ecosystems through plant photosynthetic activities (e.g., nutrient uptake and litter production) and soil freeze–thaw cycles (Groffman and Tiedje, 1989; Scott-Denton et al., 2003). The carbon (C) cycle in mid-to-high latitude forests is also expected to have more pronounced effects on global warming than in other areas of the globe (Lal, 2005). Because soil microbial communities play a major role in regulating climate feedbacks to the C cycle (Bardgett et al., 2008), predicting the impacts of seasonal changes in climate conditions on the diversity and composition of soil microbes should be a high priority for management of these forested areas (Allison and Treseder, 2008). Nevertheless, how periodic seasonal changes in environmental factors shape soil microbial communities remains poorly understood in forest ecosystems at mid-to-high latitudes.

Soil microorganisms, especially fungi and bacteria, are the main actors driving forest ecosystem functioning, given the immense diversity and abundance of these taxonomic groups. In comparison to soil fauna, fungal and bacterial communities show high respiration rates (Setälä et al., 1988) and transcription of carbohydrate-active enzymes (Žifčáková et al., 2017). For fungi and bacteria, the composition of both community members can change dramatically with the seasons because taxonomic groups of soil microbes differ in response to soil properties and plant phenology (Schmidt et al., 2007). For example, although the fungal genus *Russula* dominated during the plant growing season, the fungal genus *Mortierella* dominated during autumn and winter (Voříšková et al., 2014; Santalahti et al., 2016). In temperate forest soils, the relative abundance of the bacterial phylum *Actinobacteria* increases during winter, which can be offset by a decrease in the abundance of *Acidobacteria* and *Proteobacteria* (Kuffner et al., 2012). In comparison to soil bacteria, soil fungi tend to utilize recalcitrant organic matter in soils and plant litters, highlighting that seasons can also influence the relative abundance of fungi and bacteria through plant litter inputs and soil properties (Bardgett et al., 2005). However, because most soil microbial studies on seasonal dynamics have been conducted during distinct seasons (e.g., spring, summer, autumn, and winter; Voříšková et al., 2014; Žifčáková et al., 2017), continuous seasonal patterns in soil fungal and bacterial communities are not well understood. There is a lack of knowledge about how soil microbial communities continuously change and what the roles of environmental factors are in shaping soil fungal and bacterial communities through all seasons. Because fungi and bacteria have different physiological traits and related functional roles in forest ecosystems (Bååth and Anderson, 2003; Schneider et al., 2012), these features might create the differences in seasonal dynamics between soil fungi and bacteria. However, continuous seasonal patterns have not been explored simultaneously for both soil fungi and bacteria in forest ecosystems. In forest soils, clarifying the differences in seasonal dynamics of the diversity, taxonomic composition, and abundance between fungi and bacteria are essential for understanding microbial community assembly.

Not only seasons but also spatial variations can be important factors determining microbial diversity and community structures in forest soils (Ettema and Wardle, 2002). For soil bacteria, Fierer and Jackson (2006) found that spatial differences in community structures could be explained by soil pH, shaping biogeographical patterns. In our previous study, elevational diversity gradients of soil bacteria were controlled by the indirect effects of climate conditions, via plant functional diversity and soil properties (Shigyo et al., 2019). Even in the study of elevational diversity gradients of fungal and bacterial communities in forest soils, there was evidence that elevation has differential effects on soil fungal and bacterial communities (Peay et al., 2017). Besides, because of the uneven distribution of microbially available nutrients and plant roots, the contributions of soil depth can be higher than those of geographical differences for soil microbial communities (Eilers et al., 2012). Recently, Engelhardt et al. (2018) pointed out that drying–rewetting cycles depending on soil depth influence the diversity and community structures of soil fungi and bacteria. Clarifying whether a particular taxonomic group of microbes depends on spatial or temporal dynamics is not only essential for understanding the ecology of focal taxa, but also for the processes of microbial community assembly. Despite a growing number of studies considering both space and time that are used to explain soil microbial communities (Lazzaro et al., 2015; Siles et al., 2017), there has been no study to examine the importance of seasonal dynamics in comparison to elevation and soil depth.

Several abiotic and biotic conditions have direct contributions to both temporal and spatial dynamics of soil microbial communities in forests. First, climate conditions, particularly soil temperature (ST) and soil water content (SWC), can be crucial factors driving the seasonal dynamics of microbial communities in forest soils because of the physiological responses of microbes to climate conditions (Baldrian et al., 2013). For example, Allison and Treseder (2008) conducted soil warming experiments in boreal forests and found an increase in fungal diversity with soil ammonium and nitrate availability in response to warming and drying, where the relative abundance of thelephoroid fungi decreased while those of *Ascomycota* and *Zygomycota* increased. Second, the seasonal changes in soil microbial communities can be dependent on changes in soil chemical properties. In forest ecosystems at mid-to-high latitudes, for example, increasing the supply of inorganic nitrogen (N) at snow melting season can affect soil microbial communities (Schmidt et al., 2007). Finally, plant phenology can influence the seasonal dynamics of soil microbial communities because plants affect C and N availability for soil microbes as a result of exudation of labile C through roots and substrate input by litterfall (Bardgett et al., 2005). To identify the processes structuring the seasonal dynamics of soil microbial communities, it is necessary to consider the possible factors including climate conditions, soil properties, and plant traits.

This study aimed to clarify the seasonal dynamics of soil fungal and bacterial communities and their taxonomic differences in cool-temperate montane forests. At four soil layers in three elevation sites, soil samples were collected every month for a year. Both fungal and bacterial communities were investigated by high-throughput sequencing. In this study, we addressed the following three questions: (i) How do the soil fungal and bacterial communities change with seasons? (ii) How significant are these changes relative to those of elevation and soil depth? (iii) How are seasonal dynamics in soil fungal and bacterial communities affected by climate conditions, soil fertility, and plant phenology?

## Materials and Methods

### Study Site

The study was conducted at three elevations in cool-temperate and sub-alpine forests in the University of Tokyo Chichibu Forest (35°56′ N, 138°52′ E) in central Japan. The study area was composed of a mosaic of old-growth and secondary forest stands with a minimum age of about 50 years. The understory was sparsely covered by the dwarf bamboos *Sasa borealis* and *S. hayatae* in this area. In 2011, three survey plots (30 m × 30 m), hereafter referred to as high, middle and low elevation plots, were established at 1831.8, 1334.2, and 880.4 m above sea level (a.s.l.), respectively (Supplementary Table S1). Each of the three survey plots was divided into nine 10 m × 10 m grids, and the central grid was chosen for sampling and environmental measurements. Although the high elevation site was located in sub-alpine forests dominated by *Tsuga diversifolia*, the middle and low elevation sites were located in cool-temperate forests dominated by *Carpinus tschonoskii* (Supplementary Table S1). Because the species composition of trees in the study area is similar to that in nearby areas (Franklin et al., 1979; Shigyo et al., 2017; Umeki et al., 2018), these sites represent typical forest types widely distributed in this part of cool-temperate and sub-alpine forests. The mean annual temperature and precipitation for 15 years from 1996 to 2010 at Tochimoto (740 m a.s.l.), the nearest meteorological station, were 11.0°C and 1514.2 mm, respectively. Mean annual temperature decreases with increasing elevation at this site, while mean annual precipitation does not vary consistently with elevation across the study area.

### Field Sampling and Environmental Measurements

#### Soil Sampling and Soil Properties Measurements

At the beginning of every month from July 2016 to June 2017, three soil cores were collected with a root auger (DIK-102A-A1, Daiki Rika Kogyo, Saitama, Japan) from the central grids in three survey plots and split into four soil depths (0–5, 5–10, 10–20, and 20–30 cm). For soil and microbial analyses, soil samples of three cores at the same depth were well mixed and pooled for each month. A total of 144 soil samples (3 plots × 12 months × 4 depths) were collected. To investigate soil properties, pH, C:N ratio, anions, and cations were measured for these samples. Soil pH was measured using a glass electrode (Eutech pH700, Eutech Instruments Pty Ltd., Singapore) in a 1:2.5 soil-to-water extract. The concentrations of total C and N were measured using a CN analyzer (Sumigraph NC-22, Sumika Chemical Analysis Service Ltd., Tokyo, Japan) and then the C:N ratio of each sample was calculated for statistical analyses. The concentrations of water-soluble anions, chloride (Cl^–^), nitrite (NO_2_^–^), nitrate (NO_3_^–^), phosphate (PO_4_^3–^) and sulfate (SO_4_^2–^), and cations, sodium (Na^+^), ammonium (NH_4_^+^), potassium (K^+^), calcium (Ca^2+^) and magnesium (Mg^2+^), were measured using ion chromatography (IC 761 Compact, Metrohm AG, Herisau, Switzerland). Water-soluble anions and cations of each soil sample were extracted by sonication of 3 g of soil with 30 ml of deionized water for 20 min and then filtered using a 0.2 μm membrane filter (GL Chromatodisc 25A, GL Science, Tokyo, Japan). Anions and cations were measured using Metrosep A Supp 5 and C 4 columns (Metrohm AG). For microbial analyses, soil samples were stored at −80°C until DNA was extracted.

#### Climate Conditions

For each survey plot, ST (°C) at 10-cm depth was measured at 90-min intervals from July 2016 to June 2017, using a button-type temperature sensor (Thermochron G-type, KN Laboratories, Osaka, Japan). The mean value of ST measured during the 10 days before each soil sampling time was used as a representative value for each month. SWC (%) was measured gravimetrically for each soil sample by drying the soil at 80°C for 72 h.

#### Plant Sampling, Measurements and Canopy Conditions

At the same time as the soil sampling was conducted, leaves and shoots of under-canopy were collected from three trees in the same grids. Leaves and current-year shoots of each sample were freeze-dried for 24 h and then powdered by a bead beater-type homogenizer. Leaf C concentration (%), leaf N concentration (%), shoot C concentration (%) and shoot N concentration (%) were analyzed using the CN analyzer (Sumigraph NC-22) and then the C:N ratio of each sample was calculated for statistical analyses. For investigating the seasonal change of canopy conditions in each survey plot, hemispherical photographs were taken with a fisheye camera (Coolpix 950, Nikon; Fisheye Converter FC-E8, Nikon Corp., Tokyo, Japan) at the height of 1.3 m above the ground in the central grids. Canopy openness was calculated from photographs using SOLARCALC 7.0 (Mailly et al., 2013).

### Microbial Community Analyses

#### DNA Extraction, PCR Amplification, and Sequencing

Extraction of DNA from fresh soil samples (0.5 g) was performed using the NucleoSpin Soil DNA kit (Macherey-Nagel GmbH & Co., KG) with recommended amounts of the buffer SL2 and enhancer SX. The DNA yields were measured with Qubit dsDNA BR assay (Thermo Fisher Scientific, Waltham, MA, United States). Soil fungal communities were characterized by amplifying fragments of the internal transcribed spacer 2 (ITS2) region using the forward primer gITS7 (5′-GTGARTCATCGARTCTTTG-3′; Ihrmark et al., 2012) and the reverse primer ITS4ngs (5′-TTCCTSCGCTTATTGATATGC-3′; Tedersoo et al., 2014) on a thermal cycler (GeneAtlas G, ASTEC, Fukuoka, Japan). Soil bacterial communities were also characterized by amplifying fragments of the V4 hypervariable region of the 16S ribosomal RNA (rRNA) gene using the forward primer 515F (5′-GTGCCAGCMGCCGCGGTAA-3′; Caporaso et al., 2012) and the reverse primer 806R (5′-GGACTACHVGGGTWTCTAAT-3′; Caporaso et al., 2012) on the thermal cycler.

For both primer sets, a two-step tailed PCR method was employed for high-throughput sequencing. The first PCR reactions were carried out in 25 μl reaction mixtures containing 5 ng of soil DNA, 0.25 μM each forward and reverse primers, 12.5 μl of 2 × Gflex PCR Buffer, and 0.5 μl of Tks Gflex DNA polymerase (Takara Bio Inc., Shiga, Japan). For fungal communities, the protocol for the first PCR was 94°C for 2 min, followed by 35 cycles of 10 s at 98°C, 15 s at 56°C, and 30 s at 68°C, with a final extension at 68°C for 7 min. The first PCR protocol for bacterial 16S rRNA was 94°C for 2 min, followed by 35 cycles of 10 s at 98°C, 15 s at 50°C, and 30 s at 68°C, with a final extension at 68°C for 5 min. AMPure XP beads (Beckman Coulter, Brea, CA, United States) were used to purify the ITS2 and 16S rRNA amplicons and remove free primers and primer dimers. The first PCR products were quantified by Qubit dsDNA HS assay (Thermo Fisher Scientific, Waltham, MA, United States). The second PCR was carried out in 25 μl reaction mixtures including 10 ng of the template DNA amplified in the first PCR, 0.25 μM each forward and reverse primers for the second PCR, 2.5 μl of 2 × PCR Buffer for KOD -Multi & Epi-, and 0.5 μl of KOD -Multi and Epi- (Toyobo Co., Ltd., Osaka, Japan). In the second PCR, PCR amplification added multiplexing index sequences to the overhang adapters using a multiplex primer pair combination for each sample. The thermal cycling conditions were 94°C for 2 min, ten cycles of 98°C for 10s, 60°C for 15s, 68°C for 30 s, and final extension 68°C for 5 min. The second PCR products were cleaned using AMPure XP beads and quantified by Qubit dsDNA HS assay. Finally, all samples were pooled together in equimolar concentrations. Sequencing for fungal and bacterial communities was performed on a MiSeq platform (Illumina, San Diego, CA, United States) using 2 × 300 bp and 2 × 250 bp paired-end reads, respectively (FASMAC Co., Ltd., Kanagawa, Japan).

#### Bioinformatic Analyses

For ITS2 amplicons, the sequencing data were processed using the PIPITS 1.5.0 pipeline (Gweon et al., 2015). Briefly, the forward and reverse paired-end sequences were merged using VSEARCH (Rognes et al., 2016), and then quality-filtering was undertaken with the FASTX-Toolkit (Gordon and Hannon, 2010). The fungal ITS2 region was extracted with ITSx software (Bengtsson-Palme et al., 2013). The 97% similarity level was finally established for the operational taxonomic units (OTUs) using VSEARCH. Taxonomic assignments were conducted using the RDP classifier algorithm (Wang et al., 2007) against the UNITE fungal ITS database (Abarenkov et al., 2010). For 16S rRNA amplicons, the sequencing data were processed using QIIME v. 1.9.1 pipeline (Caporaso et al., 2010). The paired-end sequences were merged, and then quality filtered using PANDAseq (Masella et al., 2012). A closed reference-based OTU picking approach was used to cluster reads into OTUs at 97% sequence similarity using the UCLUST algorithm (Edgar, 2010). Taxonomy was assigned using the RDP classifier algorithm against the Greengenes v13_8 database (DeSantis et al., 2006). For statistical analyses of fungal and bacterial communities, sequences of each sample were rarefied to 21,307 and 19,998 sequences, respectively, based on the sample with the lowest sequencing depth.

#### Quantitative PCR Analyses

The abundance of soil fungi and bacteria was assessed by quantitative polymerase chain reaction (qPCR), using fungal ITS region primers ITS1 (5′-TCCGTAGGTGAACCTGC GG-3′; Gardes and Bruns, 1993) and 5.8s (5′-CGCTGCGTTCTT CATCG-3′; Vilgalys and Hester, 1990) and bacterial 16S rRNA encoding gene primers Eub338 (5′-ACTCCTACGGG AGGCAGCAG-3′; Lane, 1991) and Eub518 (5′-ATTACCGCG GCTGCTGG-3′; Muyzer et al., 1993), respectively. The qPCR was performed on 96 well plates using the QuantStudio 3 real-time PCR system (Applied Biosystems, Carlsbad, CA, United States). The quantification of the 16S rRNA gene for bacteria and the ITS gene for fungi to estimate the total microbial abundance was performed using 1 μl of template DNA, 10 μl KOD SYBR qPCR Mix, 0.2 μM each forward and reverse primers, 0.4 μl 50 × ROX Reference Dye, and water to adjust to a final volume of 20 μl (Toyobo). The qPCR conditions were initial denaturing at 98 for 2 min, followed by 40 cycles for 10 s at 98°C, 53°C for 10 s, 68°C for 30 s, and a final step for the melting curve. Plasmid standards for quantification of fungal ITS and bacterial 16S rRNA gene copy numbers were selected from the clone library. Plasmids standards for fungal ITS and bacterial 16S rRNA were prepared by cloning amplified genomic DNA of *Serpula himantioides* and *Pseudomonas aeruginosa*, respectively. Fungal ITS and bacterial 16S rRNA gene copy numbers were generated using regression equations relating copy numbers to the cycle threshold (*C*_t_) values. All of the qPCR reactions were run in triplicate with the DNA extracted from each sample.

### Statistical Analyses

Statistical analyses were conducted for both fungal and bacterial communities. All statistical analyses were performed using the R environment for statistical computing version 3.4.2 (R Development Core Team, 2017). Multiple linear regression analyses were performed to determine how the diversity and abundance of soil microbes varied seasonally and spatially. In these analyses, the response variables were the number of genera and gene copies of soil fungi and bacteria, and the explanatory variables were seasons, elevation, and soil depth. The variables on seasons were represented as cos(2π *d*/365) and sin(2π *d*/365), *Sc* and *Ss*, respectively, where d is the number of days counted from first soil sampling date (i.e., July 3, 2016). The coefficients of *Sc* and *Ss* can take positive or negative values depending on how the number of genera and gene copies responds to the season. In multiple linear regression analyses including both of these two variables, all days can be placed as a peak (Supplementary Figure S1). The elevation variable (*Ele*) was calculated from airborne LiDAR point cloud data (Shigyo et al., 2017). The soil depth variable (*Dep*) was treated as a numerical one; 0–5 cm = 2.5 cm, 5–10 cm = 7.5 cm, 10–20 cm = 15 cm, and 20–30 cm = 25 cm. All explanatory variables were standardized to mean zero and unit variance. Then, a likelihood ratio test was applied to evaluate the relative importance of seasons (*Sc* and *Ss*), elevation, and soil depth by comparing the model with null models without these variables. The fit of the regressions was assessed using *R*^2^ and the variance explained by each explanatory variable (Δ*R*^2^).

Finite mixtures of negative binomial regression models with an algorithm for model-based clustering were used to identify clusters in fungal and bacterial communities at the genus level and to assess the relative importance of seasons (*Sc* and *Ss*), *Ele*, and *Dep* for each cluster. This approach uses the expectation-maximization (EM) algorithm to obtain the maximum likelihood parameter estimates (Leisch, 2004), allowing the simultaneous grouping and quantification of the responses of multiple microbial genera to seasonal changes. The EM algorithm has two steps, finding the expected value of the likelihood function (E-step) and maximization of the likelihood function (M-step). Although this approach has not often been used in microbial community ecology, it is known to be effective in analyzing the niche partitioning of multiple species along environmental gradients (Dunstan et al., 2011; Ouédraogo et al., 2013). In the current study, finite mixtures with two to 20 clusters were fitted for fungi and bacteria. For each number of clusters, the EM algorithm was repeated five times with random initialization. The Bayesian information criterion (BIC) was used to determine the optimal number of clusters. To assess model fit and predictive accuracy, the percentage of explained deviance (*D*) was computed for each model: *D* = 100 × (null deviance − residual deviance)/null deviance (Guisan and Zimmermann, 2000). The explained deviance by each explanatory variable (Δ*D*) was also calculated. These models were calculated using the flexmix (Leisch, 2004) and countreg (Kleiber and Zeileis, 2016) packages running on R. Furthermore, to find an indicator genus for each cluster, we conducted the compositional indicator genus analyses using the labdsv package (Roberts, 2010). Here, an indicator value for each genus was calculated by using the mean similarity among all samples in a cluster. The *P*-value of an indicator value was calculated by comparing that value to the distribution of mean similarities for a randomly generated set with the same size.

Generalized linear models (GLM) were used to identify environmental variables, including climate conditions (ST and SWC), soil chemical properties (pH, soil C, soil N, soil C:N ratio, Cl^–^, NO_2_^–^, NO_3_^–^, PO_4_^3–^, SO_4_^2–^, Na^+^, NH_4_^+^, K^+^, Ca^2+^, and Mg^2+^), and plant traits (canopy openness, leaf C, leaf N, leaf C:N ratio, shoot C, shoot N, and shoot C:N ratio), correlated with the number of genera and gene copies, and genus level sequence counts of each cluster. In the models, principal component analysis (PCA) was applied to reduce the number of explanatory variables for soil properties and plant traits. The first principal component (PC) axes were selected for soil properties, explaining 27.0% of the total variance (Supplementary Table S2). The first PC (Soil PC1) negatively correlated with soil pH and NO_2_^–^, SO_4_^2–^, and Ca^2+^ and positively correlated with C, N, C:N ratio, NO_3_^–^, PO_4_^3–^, NH_4_^+^, and K^+^. Here, Soil PC1 is interpreted as an organic material driven fertility gradient. For plant traits, the first PC axis (Plant PC1) was positively correlated with canopy openness and shoot N and negatively correlated with leaf C, leaf N, leaf C:N ratio, shoot C and shoot C:N ratio. Plant PC1 explained 64.3% of the variance in plant traits (Supplementary Table S3). For examining the potential influence of multicollinearity, variance inflation factors (VIF) were calculated for each explanatory variable in the models, but all VIF values were <10, implying that there was no variable highly correlated with any other variables. GLMs were fitted with the Gaussian distribution for the number of genera and gene copies, and the negative binomial distribution for sequence counts of each cluster. Finally, for each model, the stepwise model selection was performed based on the Akaike information criterion (AIC), using backward selection to identify the minimum adequate model. For all GLMs, *D* and Δ*D* were calculated to assess model fit and predictive accuracy.

Additionally, multiple linear regression analyses were performed to determine how the environmental variables varied seasonally and spatially. In these analyses, the response variables were ST, SWC, Soil PC1, and Plant PC1, and the explanatory variables were seasons (*Sc* and *Ss*), *Ele*, and *Dep*.

## Results

In this study, the amplicon sequencing of soil fungal ITS2 sequences resulted in the identification of a total of 5312 OTUs with 523 genera of fungi. Soil fungal communities were dominated by *Basidiomycota*, followed by *Ascomycota* and *Mortierellomycota*. The most abundant fungal genera were *Lactarius* (*Basidiomycota*), *Russula* (*Basidiomycota*), and an unidentified genus of the order *Helotiales* (*Ascomycota*). For the amplicon sequencing of soil bacterial 16S rRNA sequences, we detected a total of 8074 OTUs with 797 genera of bacteria. Bacterial communities in soils were dominated by *Proteobacteria*, followed by *Acidobacteria* and *Actinobacteria*. The most abundant bacterial genera were an unidentified genus of the order *Ellin6513* (*Acidobacteria*), an unidentified genus of the family *Rhodospirillaceae* (*Proteobacteria*) and *Hyphomicrobiaceae* (*Proteobacteria*).

**FIGURE 1:**
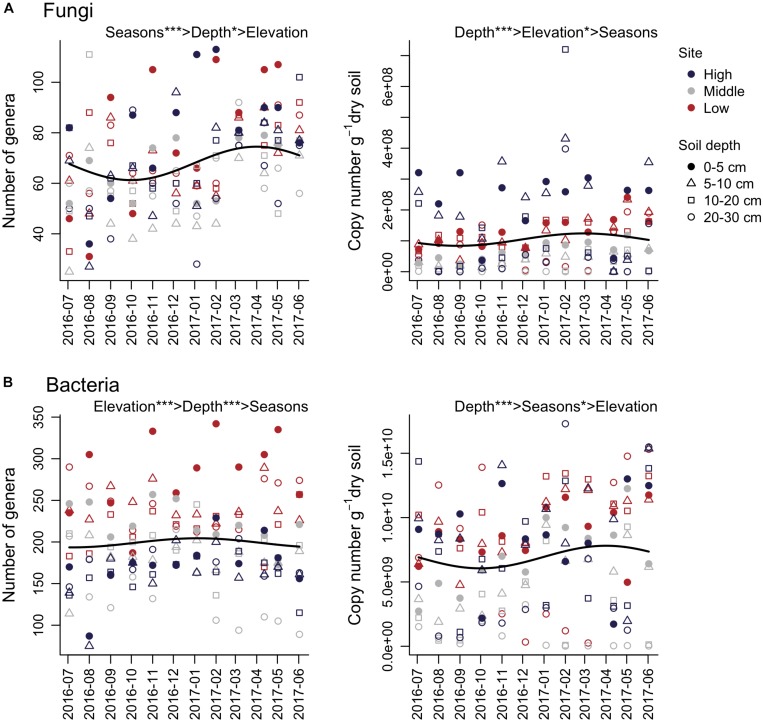
Seasonal dynamics of the number of genera and gene copies of soil fungi **(A)** and bacteria **(B)**. The time series starts on July 3, 2016. The importance of seasons (cosine and sine function with 1-year periodicity; *Sc* and *Ss*), elevation, and soil depth are denoted (e.g., Elevation > Depth shows that elevation has higher relative importance than soil depth). Solid lines represent fitted equations from multiple regression analyses based on *Sc* and *Ss*. ^∗∗∗^*P* < 0.001 and ^∗^*P* < 0.05.

Multiple regression analyses showed a significant relationship between the number of fungal genera and seasons, where the peak of the number of fungal genera was observed in April, and the minimum occurred in October (Figure 1A and Table 1). Although the number of fungal genera was negatively associated with soil depth, the relative importance of seasons was higher than that of soil depth (Table 1). The number of copies of the fungal ITS gene showed a positive association with elevation and a negative association with soil depth and *Ss*, where elevation had stronger correlations than soil depth and seasons (Table 1). For soil bacteria, the number of genera showed significant associations with elevation and soil depth, where the relative importance of elevation was the highest (Figure 1B and Table 1). The number of copies of the 16S rRNA gene had significant associations with soil depth and seasons, where the variable with the highest relative importance was soil depth (Figure 1B and Table 1). The peak of the number of bacterial 16S rRNA gene copies was apparent in April, and the minimum was observed in October (Figure 1B and Table 1). In these analyses, except for the number of bacterial genera, showed relatively low *R*^2^ (Table 1). However, the Δ*R*^2^ of the *Ss* explaining the number of fungal genera was higher than that of *Ss* explaining the number of bacterial genera (Table 1).

**TABLE 1:** Results of multiple linear regression analyses.

	***Sc***			***Ss***			***Ele***			***Dep***			***R*^2^**
	**Coefficient**	***P*-value**	**Δ*R*^2^**	**Coefficient**	***P*-value**	**Δ*R*^2^**	**Coefficient**	***P*-value**	**Δ*R*^2^**	**Coefficient**	***P*-value**	**Δ*R*^2^**	
**(A) Fungi**													
Number of genera	−0.24	0.87	0	**−6.61**	<0.001	0.13	−0.88	0.54	0	**−3.12**	0.03	0.03	0.16
Number of gene copies	−1.11E + 07	0.19	0.01	−1.67E + 07	0.05	0.02	**2.01E + 07**	0.02	0.03	**−3.62E + 07**	<0.001	0.11	0.18
**(B) Bacteria**													
Number of genera	−5.46	0.07	0.01	-0.36	0.91	0	**−32.61**	<0.001	0.42	**−13.23**	<0.001	0.07	0.50
Number of gene copies	−2.49E + 07	0.94	0	**−8.77E + 08**	0.01	0.04	−6.28E + 08	0.07	0.02	**−1.42E + 09**	<0.001	0.10	0.16

*Seasons (cosine and sine functions with 1-year periodicity; *Sc* and *Ss*), elevation (*Ele*), and soil depth (*Dep*) to explain the number of genera and gene copies of soil fungi (A) and bacteria (B). Significant slope values (*P* < 0.05) are highlighted in bold.*

**FIGURE 2:**
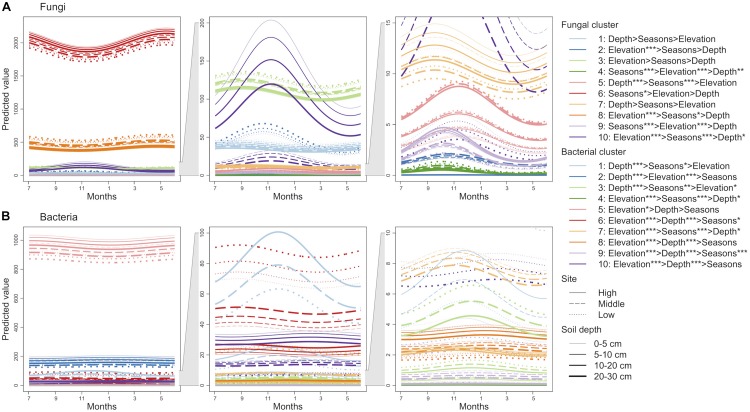
Seasonal dynamics of the predicted sequence counts of soil fungi **(A)** and bacteria **(B)** based on finite mixtures of negative binomial regression models. The time series starts on July 3, 2016. The clusters are colored differently. Solid, dashed, and dotted lines represent high, middle, and low elevation sites, respectively. Width of lines increases from shallow to deep soil depth. The importance of seasons (cosine and sine function with 1-year periodicity; *Sc* and *Ss*), elevation, and soil depth are denoted (e.g., Elevation > Depth shows that elevation has higher relative importance than soil depth). ^∗∗∗^*P* < 0.001, ^∗∗^*P* < 0.01, and ^∗^*P* < 0.05.

**FIGURE 3:**
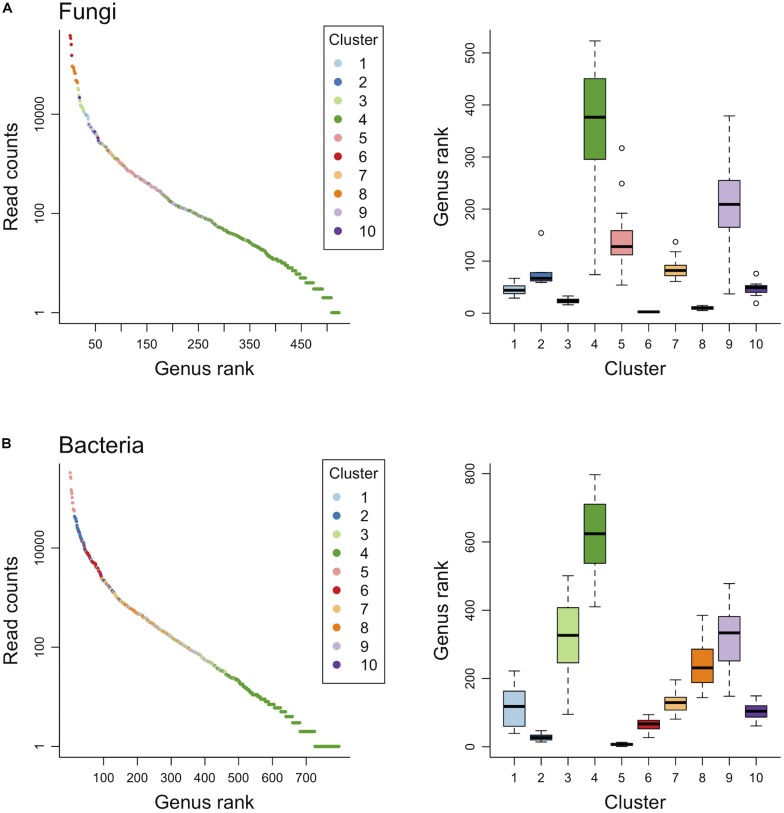
Genus level rank abundance distribution and the range of genus rank per cluster for fungal **(A)** and bacterial **(B)** communities.

Finite mixtures of negative binomial regression models showed that the fungal and bacterial sequence count data were best classified into 10 clusters (Figure 2 and Table 2). For fungi, sequence counts in clusters 2, 4, 5, 6, 8, 9, and 10 were significantly associated with seasons. The importance of seasons was higher than elevation and soil depth in clusters 4, 6, and 9 (Figure 2A and Table 2). However, fungal clusters 1, 3, and 7 were not significantly explained by seasons, elevation, or soil depth (Figure 2A and Table 2). The explained deviance was less than five percent, except for fungal clusters 2, 4, 9, and 10. The genus level sequence counts of soil bacteria in clusters 1, 3, 4, 6, 7, and 9 were significantly related to seasons (Figure 2B and Table 2). For all bacterial clusters, the relative importance of seasons was lower than elevation or soil depth (Figure 2B and Table 2). The explained deviance for bacterial clusters 2, 4, 5, and 8 was less than five percent. Furthermore, soil microbial taxa were defined by compositional indicator genus analyses for each cluster (Table 3). The indicator genera are taxa that best represent the response to the seasonal dynamics of each cluster. For both fungal and bacterial communities, the genus level rank abundance distribution was left-skewed with a few dominant genera and many rare genera (Figure 3). Notably, fungal clusters 4, 5, and 9, and bacterial clusters 3, 4, and 9 were rare while fungal clusters 6 and 8, and bacterial clusters 2 and 5 were consistently dominant.

**TABLE 2:** Results of finite mixtures of negative binomial regression models.

	***Sc***			***Ss***			***Ele***			***Dep***			***D***
	**Coefficient**	***P*-value**	**Δ*D***	**Coefficient**	***P*-value**	**Δ*D***	**Coefficient**	***P*-value**	**Δ*D***	**Coefficient**	***P*-value**	**Δ*D***	
**(A) Fungi**													
Cluster 1	0.06	0.19	0.06	0.05	0.30	0.04	–0.05	0.35	0.04	–0.07	0.12	0.09	0.22
Cluster 2	–0.12	0.16	0.20	**0.34**	<0.001	1.99	**−2.98**	<0.001	56.55	0.08	0.36	0.06	58.81
Cluster 3	0.03	0.33	0.05	0.07	0.07	0.14	–0.06	0.06	0.14	0.01	0.72	0.01	0.33
Cluster 4	–0.01	0.90	0.55	**0.81**	<0.001	4.50	**−0.29**	<0.01	0.87	0.15	0.13	0.20	6.12
Cluster 5	**−0.18**	0.01	0.28	**0.21**	<0.01	0.61	–0.02	0.76	0.00	**0.31**	<0.001	1.13	2.03
Cluster 6	0.04	0.12	0.45	–0.05	0.06	0.56	0.03	0.22	0.23	–0.01	0.61	0.04	1.29
Cluster 7	0.03	0.68	0.01	0.11	0.09	0.12	0.07	0.43	0.02	–0.10	0.13	0.10	0.24
Cluster 8	0.03	0.29	0.03	**−0.07**	0.01	0.35	**−0.13**	<0.001	1.17	0.03	0.22	0.08	1.63
Cluster 9	–0.07	0.45	0.09	**0.59**	<0.001	4.04	**0.31**	<0.001	1.16	0.05	0.57	0.01	5.31
Cluster 10	**−0.25**	0.02	2.02	**0.34**	<0.01	0.58	**1.44**	<0.001	12.98	**−0.20**	0.05	0.40	15.98
**(B) Bacteria**													
Cluster 1	**−0.17**	0.05	0.20	0.13	0.10	0.00	0.19	0.05	0.00	**1.18**	<0.001	16.76	16.96
Cluster 2	–0.02	0.21	0.04	–0.00	0.87	0.00	**0.08**	<0.001	0.70	**−0.10**	<0.001	0.77	1.52
Cluster 3	**−0.16**	<0.01	0.13	0.06	0.24	0.40	**−0.15**	0.03	0.05	**1.17**	<0.001	16.95	17.54
Cluster 4	**−0.13**	0.01	0.07	**0.15**	<0.01	0.32	**−0.56**	<0.001	4.47	0.09	0.07	0.12	4.99
Cluster 5	0.02	0.47	0.03	0.00	0.96	0.00	**0.04**	0.04	0.19	–0.03	0.22	0.07	0.29
Cluster 6	0.03	0.08	0.01	**0.04**	0.03	0.06	**−0.50**	<0.001	9.92	**0.10**	<0.001	0.53	10.52
Cluster 7	**−0.05**	0.04	0.00	**0.08**	<0.001	0.02	**−0.96**	<0.001	21.12	**−0.05**	0.02	0.08	21.22
Cluster 8	**−0.05**	0.02	0.12	–0.01	0.70	0.01	**0.24**	<0.001	1.56	**−0.07**	<0.001	0.14	1.83
Cluster 9	**−0.08**	<0.001	0.03	0.02	0.31	0.01	**−1.34**	<0.001	27.87	**−0.25**	<0.001	1.10	29.01
Cluster 10	–0.03	0.22	0.24	–0.02	0.40	0.09	**0.58**	<0.001	13.76	**−0.08**	<0.001	0.28	14.36

*Seasons (cosine and sine functions with 1-year periodicity; *Sc* and *Ss*), elevation (*Ele*), and soil depth (*Dep*) to identify clusters in fungal (A) and bacterial (B) sequence counts. Significant slope values (*P* < 0.05) are highlighted in bold. *D* represents the percentage of explained deviance, and Δ*D* shows the explained deviance by each explanatory variable.*

**TABLE 3:** List of the most reliable indicator taxa for fungal (A) and bacterial (B) clusters.

**Clusters**	**Phylum**	**Class**	**Order**	**Family**	**Genus**	**Indicator value**	***P*-value**
**(A) Fungi**							
Cluster1	*Basidiomycota*	Unidentified	Unidentified	Unidentified	Unidentified	0.39	0.01
Cluster2	*Ascomycota*	*Pezizomycotina*	*GS35*	Unidentified	Unidentified	0.40	0.01
Cluster3	*Basidiomycota*	*Tremellomycetes*	*Cystofilobasidiales*	*Cystofilobasidiaceae*	*Mrakia*	0.51	0.01
Cluster4	*Ascomycota*	*Sordariomycetes*	*Hypocreales*	*Ophiocordycipitaceae*	*Haptocillium*	0.78	0.02
Cluster5	*Ascomycota*	*Sordariomycetes*	*Xylariales*	*Apiosporaceae*	*Arthrinium*	0.21	0.01
Cluster6	*Basidiomycota*	*Agaricomycetes*	*Russulales*	*Russulaceae*	*Lactarius*	0.74	0.01
Cluster7	*Basidiomycota*	*Agaricomycetes*	*Agaricales*	*Mycenaceae*	*Xeromphalina*	0.25	0.01
Cluster8	*Basidiomycota*	*Microbotryomycetes*	*Sporidiobolales*	*Sporidiobolales*	*Rhodotorula*	0.64	0.01
Cluster9	*Ascomycota*	*Dothideomycetes*	*Pleosporales*	*Leptosphaeriaceae*	*Leptosphaeria*	0.24	0.01
Cluster10	*Basidiomycota*	*Agaricomycetes*	*Agaricales*	*Strophariaceae*	*Naucoria*	0.20	0.01
**(B) Bacteria**							
Cluster1	*WS3*	*PRR-12*	*Sediment-1*	Unidentified	Unidentified	0.34	0.01
Cluster2	*AD3*	*JG37-AG-4*	Unidentified	Unidentified	Unidentified	0.65	0.01
Cluster3	*Actinobacteria*	*Actinobacteria*	*Bifidobacteriales*	*Bifidobacteriaceae*	*Bifidobacterium*	0.26	0.01
Cluster4	*Firmicutes*	*Clostridia*	*Clostridiales*	*Veillonellaceae*	*Pelosinus*	0.50	0.01
Cluster5	*Proteobacteria*	*Deltaproteobacteria*	*Syntrophobacterales*	*Syntrophobacteraceae*	Unidentified	0.75	0.01
Cluster6	*Nitrospirae*	*Nitrospira*	*Nitrospirales*	*Nitrospiraceae*	*Nitrospira*	0.56	0.01
Cluster7	*Chloroflexi*	*Ktedonobacteria*	*JG30-KF-AS9*	*Unidentified*	Unidentified	0.50	0.01
Cluster8	*Proteobacteria*	*Alphaproteobacteria*	*Rhodospirillales*	*Acetobacteraceae*	*Acidocella*	0.41	0.01
Cluster9	*Proteobacteria*	*Deltaproteobacteria*	*Myxococcales*	*OM27*	Unidentified	0.46	0.01
Cluster10	*Chloroflexi*	*TK17*	Unidentified	Unidentified	Unidentified	0.48	0.01

*For each cluster, indicator values for each genus were calculated by using the mean similarity among all samples in a cluster. The *P*-value of an indicator value was calculated by comparing that value to the distribution of mean similarities for a randomly generated set with the same size.*

The generalized linear models showed the relationships between environmental variables and the number of genera and gene copies, and sequence counts for fungal and bacterial clusters (Table 4). The number of fungal and bacterial genera showed a positive association with Soil PC1 and Plant PC1. The number of bacterial genera was negatively related to SWC. For both fungi and bacteria, the number of gene copies had negative relationships with ST and SWC and was positively associated with Soil PC1. In addition, the number of fungal ITS gene copies was negatively correlated with Plant PC1 (Table 4). SWC was the most important factor for explaining the number of bacterial genera and gene copies. However, for soil fungi, the relative importance of Soil PC1 was higher than that of ST, SWC, and Plant PC1. In terms of the most significant variable for each cluster, ST was positively associated with sequence counts in fungal clusters 1 and 3. SWC was positively associated with sequence counts in fungal cluster 7 and bacterial clusters 3 and 8 although it had a negative relationship with bacterial sequence counts in cluster 5. For fungal cluster 10 and bacterial cluster 2, sequence counts were positively related to Soil PC1. Soil PC1 was also negatively associated with fungal sequence counts in cluster 5. For fungi and bacteria, several clusters had strong relationships with Plant PC1, where fungal cluster 9 and bacterial cluster 10 had negative associations but fungal clusters 2 and 8, and bacterial clusters 4, 6, 7, and 9 had positive relationships. No relationship with environmental variables was detected for sequence counts in fungal cluster 6 (Table 4). In these models, the explained deviance for fungal clusters 1 and 3 to 9, and bacterial clusters 2, 4, 5 and 8 was less than five percent.

**TABLE 4:** Results of backward selection of generalized linear models using environmental variables, soil temperature (ST), soil water content (SWC), and principal component (PC) axes of soil chemical properties and plant traits, to explain the number of genera and gene copies, and sequencing counts for each cluster as identified by finite mixture modeling.

	**ST (°C)**		**SWC (%)**		**Soil PC1**		**Plant PC1**		***D***
	**Coefficient**	**Δ*D***	**Coefficient**	**Δ*D***	**Coefficient**	**Δ*D***	**Coefficient**	**Δ*D***	
**(A) Fungi**									
Number of genera	–	–	–	–	4.86	3.69	2.87	1.91	5.61
Number of gene copies	−2.21E + 07	5.26	−3.20E + 07	7.70	5.82E + 07	7.35	−2.04E + 07	2.09	22.40
Cluster 1	0.06	0.07	–	–	–	–	–	–	0.07
Cluster 2	1.16	2.15	–1.24	35.75	–0.60	0.66	2.13	13.17	51.73
Cluster 3	0.05	0.19	–	–	–0.05	0.09	–	–	0.27
Cluster 4	0.32	2.94	–	–	–0.25	0.04	–0.23	0.02	2.96
Cluster 5	–0.17	0.00	–	–	–0.33	0.54	–0.16	0.17	0.71
Cluster 6	–	–	–	–	–	–	–	–	–
Cluster 7	0.13	0.02	0.17	0.27	–	–	–	–	0.29
Cluster 8	–	–	–	–	–0.06	0.81	0.09	0.49	1.29
Cluster 9	–	–	–	–	–	–	–0.55	2.85	2.85
Cluster 10	–0.35	2.65	–	–	0.93	7.15	–0.74	3.89	13.70
**(B) Bacteria**									
Number of genera	–	–	–31.06	17.20	19.95	2.67	15.27	6.95	26.82
Number of gene copies	−6.73E + 08	1.37	−3.45E + 09	16.08	3.23E + 09	2.73	–	–	20.18
Cluster 1	–	–	2.02	0.00	–1.86	10.71	0.47	1.54	12.25
Cluster 2	–	–	–	–	0.12	1.64	–0.03	0.06	1.70
Cluster 3	0.19	0.19	1.90	0.01	–1.70	12.02	0.57	0.00	13.39
Cluster 4	0.30	0.49	0.16	1.29	–0.18	0.14	0.45	1.51	3.43
Cluster 5	–	–	–0.09	0.00	0.07	0.16	–0.05	0.21	0.37
Cluster 6	0.23	1.18	–	–	–0.13	4.83	0.39	4.52	10.54
Cluster 7	0.51	1.59	–	–	0.09	3.77	0.87	11.07	16.42
Cluster 8	–0.04	0.37	0.11	1.40	0.09	0.09	–0.10	0.19	2.05
Cluster 9	0.48	1.23	–0.71	9.45	0.55	1.60	0.98	6.33	18.60
Cluster 10	–0.20	2.87	–	–	0.25	7.53	–0.36	3.75	14.15

**D* represents the percentage of explained deviance, and Δ*D* shows the explained deviance by each explanatory variable.*

Also, for multiple regression analyses, ST had a positive relationship with *Sc* and *Ss*, but was negatively associated with *Ele* (Supplementary Table S4). SWC was negatively related to *Sc* and *Dep* although it had a positive association with *Ele* (Supplementary Table S4). Soil PC1 was negatively correlated with *Ss* and *Dep*, but positively correlated with *Ele* (Supplementary Table S4). Plant PC1 was negatively associated with *Sc, Ss*, and *Ele* (Supplementary Table S4).

## Discussion

The seasonal dynamics of the observed diversity and abundance of soil fungi and bacteria showed various patterns. In this study, the patterns of diversity and abundance did not synchronize between fungi and bacteria. The observed fungal diversity showed seasonally larger fluctuation than bacterial diversity, as the coefficient of determination of the seasonal variable (i.e., *Ss*) for fungal diversity was more significant than that for the bacterial diversity. These findings suggest that the seasonal dynamics of fungal and bacterial communities are caused by different processes in forest soils, which is consistent with previous studies showing the relationships between seasonal changes in environmental factors and community structures of belowground microbes (Berg et al., 1998). For example, Buckeridge et al. (2013) found that soil fungal biomass was at least double that of bacteria during winter seasons at high latitudes, implying that fungi and bacteria might have different roles in biogeochemical cycles. One possible reason that soil fungi and bacteria have different seasonal diversity patterns is that soil fungi exhibit a narrower range of physiologies than do bacteria. Indeed, soil fungi are all heterotrophs, whereas soil bacteria can be photoautotrophs, heterotrophs or chemoautotrophs (Waid, 1999; Lladó et al., 2017). However, the results of the present study show that the abundance of soil fungi is stable across seasons although the diversity of soil fungi was characterized best by seasons rather than by elevation and soil depth. The mycelial network, which is highly conservative in terms of nutrient use (Boddy, 1999), might contribute to maintaining the abundance of soil fungi across seasons. Our results suggest that these differences between fungi and bacteria in response to seasons could be determined by environmental factors such as climate conditions, soil properties, and plant traits.

Climate conditions, soil properties, and plant traits, reflecting temporal variations, can have direct effects on soil microbial communities (Bardgett et al., 2005; Baldrian et al., 2013; Buscardo et al., 2018). The observed patterns show that the diversity and abundance of soil fungi are associated with soil fertility and that soil bacterial diversity and abundance are closely related to soil water contents. The results imply that the contribution of these factors to soil fungi and bacteria can result in distinct seasonal patterns. Indeed, Berg et al. (1998) investigated seasonal belowground fungal and bacterial biomass and found that water content and temperature were critical factors. Besides climate conditions, soil organic matter and pH clearly explained seasonal dynamics of soil microbial communities, as shown in previous studies (Siles et al., 2017; Buscardo et al., 2018). For example, in their study on the seasonal dynamics of soil microbial communities along elevational gradients in mixed deciduous and coniferous forests, Siles et al. (2017) found that soil fungal communities were related to the seasonal dynamics of the chemical composition of soil organic matter. Because the soil variable, reflecting K^+^ and PO_4_^3–^, explained the observed patterns of fungal and bacterial communities (Table 4 and Supplementary Table S2), the current study also indicated that both the spatial and seasonal dynamics of water-soluble ions can shape soil microbial communities. For microorganisms, K^+^ is necessary as a regulator of both cytoplasmic pH and cell turgor (Booth, 1985) and PO_4_^3–^ is involved in the control of energy metabolism and cell structures (Bergkemper et al., 2016). Overall, our results highlight differing drivers for seasonal dynamics of fungal and bacterial communities in cool-temperate forest soils. The observed bacterial diversity and abundance were largely explained by elevation and soil depth rather than seasons, implying that spatial differences rather than seasonal dynamics are more important in determining soil bacterial community structures.

Elevation is a crucial factor in regulating soil microbial communities (Peay et al., 2017; Shigyo et al., 2019). Although soil fungal communities showed large seasonal dynamics in this study, elevation was also important in determining soil microbial communities. Specifically, the observed diversity of soil bacteria decreased while fungal abundance increased with increasing elevation (Table 1). Elevated soil C:N ratio at the higher elevations might have contributed to these observed patterns. Indeed, high soil C:N ratio is often related to fungal-dominated communities (Fierer et al., 2009). Litter at higher elevations is typically more recalcitrant because of increased nutrient limitation and leaf thickness (Bruijnzeel and Veneklaas, 1998). These patterns could favor soil fungi, which can decompose more recalcitrant organic matter than soil bacteria (Schneider et al., 2012). Besides elevation, the difference in soil depth explained the abundance of both soil fungi and bacteria (Figure 1 and Table 1), leading to lower diversity and abundance at deeper depths. According to the relationships between soil depth and environmental variables (Supplementary Table S4), the influence of soil water contents that change with soil depth are considerable. In experimental studies, soil water conditions along soil depth are important factors determining soil bacterial diversity and community structures (Wang and Or, 2013; Engelhardt et al., 2018). This study highlights the importance of soil water contents for soil bacterial communities, which is consistent with previous studies (Eilers et al., 2012).

At the genus level, the taxonomic composition of both observed fungi and bacteria showed notable seasonal dynamics. The observed fungal clusters with high sequence counts demonstrated clear seasonal dynamics. For example, the ectomycorrhizal genus *Lactarius* was abundant during the plant growing season (cluster 6 in Figure 2A), which is consistent with a previous study showing seasonal changes of soil fungal communities in boreal forest ecosystems (Santalahti et al., 2016). However, the cluster was not associated with the observed plant traits (Table 4), which implies that unconsidered variables such as phenology of root growth might shape seasonal patterns of abundant fungal communities. Although bacterial clusters with high sequence counts were seasonally stable (clusters 2 and 5 in Figure 2B), the subdominant cluster represented by *Nitrospira* showed seasonal dynamics (cluster 6 in Figure 2B). Similarly, rare microbial groups (clusters 4 and 9 in Figure 2A and clusters 3 and 4 in Figure 2B) fluctuated seasonally more than dominant ones, implying that an increase in the compositional variety of soil organic matter through litterfall might facilitate growth of diverse rare microbes. Indeed, plant phenology can, directly and indirectly, affect the seasonal dynamics of soil microbial communities because plants influence C and N availability for soil fungi and bacteria as a result of exudation of labile C through the roots and substrate input by litterfall (Bardgett et al., 2005). According to the differences in the range of physiologies between soil fungi and bacteria, soil fungi are more dependent on fixed sources and environments than bacteria and might not have many available niches across seasons. Importantly, the seasonal dynamics of both dominant and rare taxonomic groups were different between fungal and bacterial communities. Although this study does not consider the interactions between soil fungal and bacterial communities, such different seasonal dynamics imply that the seasonal assembly processes are fundamentally different between soil fungal and bacterial communities.

Soil microbial communities are incredibly diverse and often contain many rare taxa. Recently, these microbial taxonomic groups have been named conditionally rare taxa (CRT; Shade and Gilbert, 2015). Because CRT can explain up to 97% of temporal dynamics in microbial community structures (Shade et al., 2014), a better understanding of CRT might provide a more complete picture of microbial community ecology and ecosystem functioning (Shade and Gilbert, 2015). Indeed, Aanderud et al. (2015) found that a soil-rewetting event resuscitated bacterial CRT and reduced the net production of methane, highlighting the contribution of rare microbial taxa to ecosystem functioning. In the current study, because the taxonomic groups were composed of genera with high rank, such as fungal clusters 4 and 9, and bacterial clusters 3 and 4, and they showed remarkable seasonal patterns, these groups were considered to be CRT. Among them, *Pelosinus*, detected as an indicator genus in cluster 4, is known as an iron-reducing bacterium (Hansel et al., 2008), which is consistent with other studies that have shown that rare microbial taxa exhibit unique functions and biogeographical patterns along environmental gradients (e.g., Gies et al., 2014). Furthermore, *Haptocillium*, an indicator genus in fungal cluster 4, are endoparasites of nematodes and *Bifidobacterium*, an indicator genus in bacterial cluster 3, are beneficial gut microbiota. These results imply that the seasonal dynamics of animal communities in forests might contribute to unexplained variations of seasonality of these microbial taxa. However, because seasonal patterns of CRT clusters were not synchronized with those of the number of genera (Figures 1, 2 and Supplementary Table S5), CRT might not account for the seasonal dynamics of the overall diversity. Therefore, whether CRT play a significant role in forest ecosystems and microbial communities is still controversial. Future research needs to clarify how seasonal changes in CRT contribute to the whole microbial diversity and how the functions of CRT contribute to forest ecosystems.

Given the magnitude of climate change predicted for soil ecosystems in mid-to-high latitudes forests, it is crucial to identify whether the seasonal dynamics of soil microbial communities in these forests are unique. The observed seasonal patterns of the dominant fungal taxa (i.e., *Lactarius*) tended to be similar to those of temperate (Voříšková et al., 2014) and boreal (Santalahti et al., 2016) forests in other regions. However, the seasonal patterns of subdominant and rare fungal taxa in those forests showed a different tendency in comparison to the results from the current study. In contrast to soil fungi, Kuffner et al. (2012) found an increase of the rare phylum *Nitrospira* in summer in a temperate montane forest, which is consistent with our results. However, there are no studies focusing on the seasonal dynamics of dominant and rare bacterial taxa in mid-to-high latitude forests (e.g., Schmidt et al., 2007; Žifčáková et al., 2017). These differences between the current and other studies can be explained by differences in climate conditions. In colder regions, seasonal snow cover might have the potential to influence soil microbial communities, resulting in the distinct seasonal patterns of taxonomic composition of soil microbes (Schmidt et al., 2007). However, snowfall seldom remains without melting in this study area (Franklin et al., 1979; Maruta et al., 1997), which might result in no changes in microbial community composition in the winter season. Our findings suggest that the rapid decline of soil temperature without snow cover shapes the unique seasonality of soil microbial communities, including both dominant and rare taxa.

This study provides the first comprehensive analysis of the seasonal and spatial dynamics of soil microbial communities in cool-temperate montane forests. Our findings were that: (i) the seasonal dynamics of the diversity and abundance of soil microbes was distinguished between fungi and bacteria, where the diversity and abundance of soil fungi were explained by soil fertility but those of soil bacteria were associated with soil water contents; (ii) the relative importance of seasons to soil fungal communities tended to be higher than that of elevation and soil depth, although soil depth clearly explained the abundance and taxonomic composition of soil fungi and bacteria; and (iii) seasonal dynamics of both abundant and rare groups were different between fungal and bacterial taxonomic compositions, and these differences were primarily explained by climate conditions, soil fertility, and plant phenology. These results imply that the contribution of seasonal changes in environmental factors to microbial communities might be equal to or greater than the effects of spatial heterogeneity of those factors. However, further studies are needed to determine what environmental factors affect the seasonal patterns of soil microbial communities, because the explanatory power of our models for most of the microbial taxa was relatively low. Overall, the presented results provide insight into the influences of environmental changes on soil fungal and bacterial communities exerted via seasonal dynamics of aboveground–belowground components and could serve to guide future studies on soil microbial ecology for improved forest ecosystem performance.

## Data Availability

Raw sequencing data are deposited in the Sequence Read Archive on the National Center for Biotechnology Information under BioProject accession number PRJDB8049.

## Author Contributions

NS, KU, and TH conceived and designed the study. NS performed the fieldwork and molecular analyses. NS and TH performed the bioinformatic analyses and led the writing of the manuscript. NS and KU conducted the statistical analyses. All authors contributed critically to the drafts and gave final approval for publication.

## Conflict of Interest Statement

The authors declare that the research was conducted in the absence of any commercial or financial relationships that could be construed as a potential conflict of interest.
