# Supplementary material for: Seasonal Dynamics of Soil Fungal and Bacterial Communities in Cool-Temperate Montane Forests
Source: Front Microbiol. 2019 Aug 23;10:1944. doi: 10.3389/fmicb.2019.01944 (PMC6716449; doi:10.3389/fmicb.2019.01944)
Supplement: Supplementary file 1 [file Data_Sheet_1.PDF]

Supplementary Material

Seasonal Dynamics of Soil Fungal and Bacterial Communities in Cool-Temperate Montane Forests*

*** Correspondence:** Nobuhiko Shigyo, shigyo@uf.a.u-tokyo.ac.jp

1 Supplementary Data

Figure S1. Hypothetical seasonal patterns of the diversity and abundance of soil microbes as cosine and sine functions in multiple linear regression analyses. The model was as follows: y = $a_1 \cos \theta + a_2 \sin \theta + INT$, where a_1 and a_2 are parameters to be estimated in the regressions; *INT* is an intercept. Then, $\theta = 2\pi\ d/365$, where *d* is the number of days counted from the first sampling date. The regression plane can be represented three-dimensionally (gray circle in panel A). If y reaches the maximum and minimum, θ becomes symmetrical about the origin 0. In panel B, the random number following the Gaussian distribution is applied to a_1 and a_2. Importantly, this regression analyses show that all days can be placed as a peak using cosine and sine functions.

Table S1. Locations and main characteristics of the study plots.

Table S2. Pearson correlations between soil properties and the first principal component (PC) axes of principal components analysis (PCA). The total explained variance of the PC is 27.0%. Significant correlations for each soil property are represented as the characteristic variables and highlighted in bold.

Table S3. Pearson correlations between plant traits and the first principal component (PC) axes of principal components analysis (PCA). The total explained variance of the PC is 64.3%. Significant correlations for each plant trait are represented as the characteristic variables of each PC axis and highlighted in bold.

Table S4. Results of multiple linear regression analyses using seasons (cosine and sine functions with one-year periodicity; *Sc* and *Ss*), elevation (*Ele*), and soil depth (*Dep*) to explain environmental valuables, soil temperature (ST), soil water content (SWC) and principal component (PC) axes of soil properties and plant traits. Significant slope values (P <0.05) are highlighted in bold.

Table S5. Correlation matrices of the predicted sequence counts of fungal (A) and bacterial (B) clusters. Significant slope values (P <0.05) are highlighted in bold.

2 Supplementary Figure and Tables

2.1 Supplementary Figure

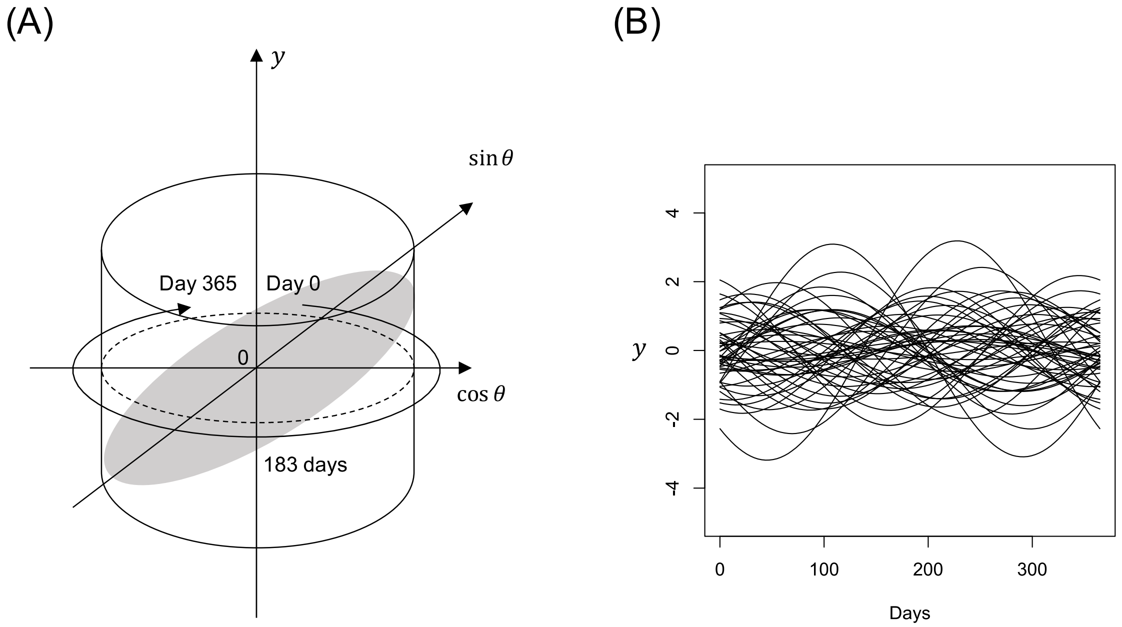

Figure S1. Hypothetical seasonal patterns of the diversity and abundance of soil microbes as cosine and sine functions in multiple linear regression analyses. The model was as follows: y = a_1 cos θ + a_2 sin θ + *INT*, where a_1 and a_2 are parameters to be estimated in the regressions; *INT* is an intercept. Then, $\theta = 2\pi\, d/365$, where *d* is the number of days counted from the first sampling date. The regression plane can be represented three-dimensionally (gray circle in panel A). If y reaches the maximum and minimum, θ becomes symmetrical about the origin 0. In panel B, the random number following the Gaussian distribution is applied to a_1 and a_2. Importantly, this regression analyses show that all days can be placed as a peak using cosine and sine functions.

2.2 Supplementary Tables

Table S1. Locations and main characteristics of the study plots.

	High elevation plot	Middle elevation plot	Low elevation plot
Coordinates	N 35° 54' 53.7"<br>E 138° 47' 51.7"	N 35° 55' 2.6"<br>E 138° 49' 3.8"	N 35° 55' 10.1"<br>E 138° 49' 53.4"
Elevation (m a.s.l.)	1831.8	1334.2	880.4
Slope (°)	30.8	19.6	28.1
Aspect	NW	S	E
Vegetation	Coniferous forest	Mixed deciduous forest	Mixed deciduous forest
Basal Area (m^2/ha)	47.2	31.4	32.1
Dominant tree species	*Tsuga diversifolia* (81.4 %)<br>*Betula corylifolia* (5.1 %)<br>*Acer austral* (4.1 %)	*Acer japonicum* (16.5 %)<br>*Betula maximowicziana* (13.4 %)<br>*Acer rufinerve* (10.6 %)	*Carpinus tschonoskii* (26.1 %)<br>*Ostrya japonica* (10.4 %)<br>*Acer amoenum* (8.4 %)

Table S2. Pearson correlations between soil properties and the first principal component (PC) axes of principal components analysis (PCA). The total explained variance of the PC is 27.0%. Significant correlations for each soil property are represented as the characteristic variables and highlighted in bold.

Soil chemical properties	Soil PC1
pH	**−0.72**
C (%)	**0.94**
N (%)	**0.87**
C:N ratio	**0.76**
Cl^-	0.13
NO_2^-	**−0.34**
NO_3^-	**0.18**
SO_4^{2-}	**−0.18**
PO_4^{3-}	**0.41**
Na^+	−0.10
NH_4^+	**0.25**
K^+	**0.47**
Ca^{2+}	**−0.38**
Mg^{2+}	0.05

Table S3. Pearson correlations between plant traits and the first principal component (PC) axes of principal components analysis (PCA). The total explained variance of the PC is 64.3%. Significant correlations for each plant trait are represented as the characteristic variables of each PC axis and highlighted in bold.

Plant traits	Plant PC1
Canopy openness	**0.61**
Leaf C (%)	**−0.88**
Leaf N (%)	**−0.33**
Leaf C:N ratio	**−0.96**
Shoot C (%)	**−0.81**
Shoot N (%)	**0.89**
Shoot C:N ratio	**−0.93**

Table S4. Results of multiple linear regression analyses using seasons (cosine and sine functions with one-year periodicity; *Sc* and *Ss*), elevation (*Ele*), and soil depth (*Dep*) to explain environmental valuables, soil temperature (ST), soil water content (SWC) and principal component (PC) axes of soil properties and plant traits. Significant slope values (P <0.05) are highlighted in bold.

	Sc		*Ss*		*Ele*		*Dep*	
	Coefficient	*P*-value	Coefficient	*P*-value	Coefficient	*P*-value	Coefficient	*P*-value
ST (°C)	**4.61**	<0.001	**3.44**	<0.001	**−1.92**	<0.001		
SWC (%)	**−1.82**	<0.001	−0.83	0.069	**8.31**	<0.001	**−4.34**	<0.001
Soil PC1	−0.10	0.150	**−0.23**	0.001	**1.33**	<0.001	**−0.99**	<0.001
Plant PC1	**−0.73**	<0.001	**−0.61**	<0.001	**−1.61**	<0.001		

Table S5. Correlation matrices of the predicted sequence counts of fungal (A) and bacterial (B) clusters. Significant slope values (*P* <0.05) are highlighted in bold.

(A) Fungi

	Cluster 1	Cluster 2	Cluster 3	Cluster 4	Cluster 5	Cluster 6	Cluster 7	Cluster 8	Cluster 9
Cluster 2	**0.38**								
Cluster 3	**0.56**	**0.72**							
Cluster 4	**0.33**	**0.57**	**0.84**						
Cluster 5	**−0.55**	**0.19**	**0.31**	**0.56**					
Cluster 6	**−0.12**	**−0.60**	**−0.68**	**−0.78**	**−0.62**				
Cluster 7	**0.51**	**−0.34**	**−0.09**	**0.09**	**−0.42**	**0.15**			
Cluster 8	**0.16**	**0.68**	**0.52**	**0.12**	**0.06**	**−0.26**	**−0.76**		
Cluster 9	**−0.08**	**−0.35**	0.00	**0.37**	**0.36**	**−0.20**	**0.63**	**−0.77**	
Cluster 10	**−0.26**	**−0.53**	**−0.58**	**−0.26**	**−0.07**	**0.33**	**0.61**	**−0.84**	**0.69**

(B) Bacteria

	Cluster 1	Cluster 2	Cluster 3	Cluster 4	Cluster 5	Cluster 6	Cluster 7	Cluster 8	Cluster 9
Cluster 2	**−0.55**								
Cluster 3	**0.90**	**−0.74**							
Cluster 4	0.02	**−0.71**	**0.31**						
Cluster 5	**−0.34**	**0.90**	**−0.59**	**−0.88**					
Cluster 6	0.03	**−0.78**	**0.33**	**0.96**	**−0.89**				
Cluster 7	**−0.19**	**−0.57**	**0.07**	**0.94**	**−0.76**	**0.94**			
Cluster 8	**−0.08**	**0.84**	**−0.34**	**−0.89**	**0.90**	**−0.95**	**−0.86**		
Cluster 9	**−0.29**	**−0.41**	**−0.10**	**0.83**	**−0.63**	**0.82**	**0.97**	**−0.75**	
Cluster 10	0.02	**0.75**	**−0.24**	**−0.88**	**0.88**	**−0.92**	**−0.86**	**0.98**	**−0.77**
